# Prospects of Noncoding RNAs in Hepatocellular Carcinoma

**DOI:** 10.1155/2018/6579436

**Published:** 2018-07-26

**Authors:** Huaixiang Zhou, Qiuran Xu, Chao Ni, Song Ye, Xiaowu Xu, Xiaoge Hu, Jiahong Jiang, Yeting Hong, Dongsheng Huang, Liu Yang

**Affiliations:** ^1^Key Laboratory of Tumor Molecular Diagnosis and Individualized Medicine of Zhejiang Province, Zhejiang Provincial People's Hospital, People's Hospital of Hangzhou Medical College, Hangzhou, Zhejiang 310014, China; ^2^Key Laboratory of Gastroenterology of Zhejiang Province, Zhejiang Provincial People's Hospital, People's Hospital of Hangzhou Medical College, Hangzhou, Zhejiang 310014, China; ^3^Department of General Surgery, Zhejiang Provincial People's Hospital, People's Hospital of Hangzhou Medical College, Hangzhou, Zhejiang 310014, China; ^4^Division of Hepatobiliary and Pancreatic Surgery, Department of Surgery, The First Affiliated Hospital, School of Medicine, Zhejiang University, Hangzhou, Zhejiang 310003, China; ^5^Department of Second Clinical Medical College, Zhejiang Chinese Medicine University, Hangzhou, Zhejiang 310053, China; ^6^Hangzhou Medical College, Hangzhou, Zhejiang 310014, China

## Abstract

Hepatocellular carcinoma (HCC) is a global health problem and one of the most common malignant tumors. Recent studies have shown that noncoding RNAs (ncRNAs) contribute to the pathogenesis of hepatocellular carcinoma (HCC). These RNAs may be involved in a variety of pathological processes such as cell proliferation, apoptosis, angiogenesis, invasion, and metastasis. In addition, abnormal expression of ncRNAs in HCC may provide potential prognostic or diagnostic biomarkers. This review provides an overview of the role and potential applications of ncRNAs, miRNAs, lncRNAs, circRNAs, and snoRNAs in liver cancer.

## 1. Introduction

Hepatocellular carcinoma (HCC) is one of the most common malignant tumors and a global health problem [1]. Like many other cancers, HCC is characterized by the involvement of multiple gene networks and the imbalance of signaling pathways [2, 3]. These genetic dysregulations involve protein-coding genes and noncoding RNA (ncRNA) genes [4]. Although the former has been the focus of research, the latter has only recently been recognized as playing a role in the pathological processes implicated in HCC [5]. Interestingly, the vast majority of the human genome is transcribed into ncRNA, while less than 2% of the genome directly encodes for proteins [6]. Noncoding RNA is a functional RNA that is not translated into protein [7].

NcRNAs include ribosomal RNA (rRNA), transfer RNA (tRNA), and small nuclear ribonucleic acids (snRNA) that process pre-mRNA, small nucleolar RNA (snoRNA), piwi-interacting RNA (piRNA), microRNA (miRNA), long noncoding RNA (lncRNA), etc. [7]. The common characteristic of these RNA is their ability to exercise their biological functions at the level [8]. Prior work has generally focused on protein-coding genes, with little focus on ncRNA function, often dismissed as nothing more than transcriptional noise [9]. However, recent studies have discovered that ncRNAs play an important role in many biological processes. Research in the field of ncRNA has shown an explosive growth in recent years since their functional role has been recognized [10–12].

With the development of high-throughput sequencing technology, many ncRNAs have been characterized as functional molecules that play an important role in various biological processes and pathological states [13–15]. In the field of hepatocellular carcinoma, some key ncRNAs have been identified as participants in the pathophysiology of the disease [16, 17]. Thousands of universally transcribed ncRNAs have been identified, and these transcripts greatly outnumber those of protein-coding mRNAs [18]. In addition, some ncRNAs show significant evolutionary conservation, indirectly supporting their functional roles [19, 20]. For example, miRNA and lncRNAs regulate different biological and pathological processes, such as tumor occurrence [21–24].

## 2. Abnormal Expression miRNAs in HCCs and Serum of Patients with Hepatocellular Carcinoma

MicroRNA (miRNA) is a type of 20-24nt long biologically functional, small molecule that is highly conserved and provides negative regulation [25]. Since their discovery in 1993 in* Caenorhabditis elegans*, their important roles continue to be described [26, 27]. Their main functions are on the transcription of regulatory proteins encoded by gene expression [28–30]. Liver cancer is both common and lethal, mainly due to ineffective treatment options [31, 32]. MiRNAs regulate protein synthesis and could either be therapeutic agents or targets for intervention [33–35]. It is clear that miRNA plays a role in proliferation, persistence, invasion, metastasis, and prognostic indicators of hepatocellular carcinoma [36–39]. In liver cancer tissue, there are many abnormally expressed miRNAs, such as miR-224, miR-221, and miR-21 that influence hepatocellular carcinoma [40–42]. miR-224 has been shown to target HOXD10 RNA and enhance both PAK4-mediated phosphorylation and MMP-9 to promote the invasion and metastasis of cancer cells [43]. MiR-224 can inhibit SMAD4 expression and promote cell proliferation [44]. Also, miR-224 can target ppp2r1b leading to excessive activation of the AKT signaling pathway, increasing the risk of liver cancer [45]. MiR-221 can target cell cycle kinase inhibitory proteins p27 and p57, thereby promoting the progression of HCC cell cycle [46, 47]. At the same time, miR-221 can interfere with the mTOR signaling pathway by inhibiting another target DDIT4, thus promoting tumor development [48]. MiR-221 expression levels can be raised by HCV infection, a process that relies on the activation of NF-*κ*B signaling [49]. MiR-221 can also target SOCS1 and SOCS3, enhancing the activation of the downstream interferon signaling pathway, thus enhancing the effectiveness of interferon against HCV [50].

In HCC cells, miR-21 can target MAP2K3 and promote the proliferation of cancer cells [51]. In parallel, miR-21 can inhibit PDCD4, thus activating the expression of downstream c-Jun, MMP-2, and MMP-9. Furthermore, AP-1 can modulate the transcription of miR-21 in a positive feedback loop, further increasing the risk of liver cancer invasion and metastasis [52]. The role of abnormally expressed miRNAs in HCCs and in the serum of HCC patients is listed in Table 1. Clinical data has shown that miR-21 expression in tumors of HCC patients is relatively high [85]. The response to postoperative IFN-Α/5-FU combined treatment is poor [86], suggesting that miR-21 may be a potential molecular marker for prognostic judgment and treatment of HCC. There are many abnormally expressed miRNAs in the serum of HCC patients. Among them, miR-16, let-7f, miR-21, miR-139, miR-101, miR-122, and miR-1 show reduced expression [87–90]. High expression of mir-17-5p may be indicative of HBV (Hepatitis B virus) and the recurrence of HCC [90]. In HCV-infected sera from HCC patients, the specific low expression of mir-30c-5p, mir-223-3p, mir-302c-5p, and mir-17-5p, as well as the specific high expression of miR-221, also suggest potential biomarkers indicating the recurrence of HCC [91, 92].

## 3. Roles of lncRNAs in HCC

Ten of thousands of lncRNAs are transcribed in humans [93]. Importantly, detecting their targets is challenging in contrast to miRNA [94]. Indeed, lncRNAs often need to form complex secondary and tertiary structures to predict targets [95].

With the development of large scale parallel sequencing technology, lncRNA has been shown to play an important role in the development of human HCC [96]. So far, many HCC-related lncRNA disorders, such as HULC, HOTAIR, MALAT1, and H19, have been used as predictive biomarkers for human disease diagnosis or prognosis [97, 98]. Furthermore, there is strong evidence that lncRNAs are associated with HCC via many signaling pathways. MyD88 levels were found to be elevated in multiple solid tumors, especially HCC [99]. Many ncRNAs, through a variety of mechanisms, regulate the location of binding sites of protein-coded genes. The abnormal increase in the expression of a new long noncoding Myd88 RNA (lnc-Myd88) is associated with HCC [66]. Chip analysis showed that lnc-Myd88 could increase Myd88 expression by enhancing the acetylation of H3K27 at the Myd88 gene promoter region, leading to the activation of NF-*κ*B and PI3K/AKT signaling pathways [66]. Thus, lnc-Myd88 may be a new diagnostic and therapeutic target for HCC. Various examples of lncRNAs regulating signaling pathways in HCC are listed in Table 2. Hence, targeting these lncRNAs in combination with other therapeutic agents could have therapeutic potential for HCC.

In addition, a new concept suggests that lncRNAs could be used as a protein scaffold close together to form ribose nuclear proteins [100]. However, only a few scaffolding lncRNAs have been identified and the broad extent of this function is unknown. LncRNAs participate in a variety of biological processes and play an important role in various human diseases, including fibrosis diseases, liver diseases, and rare human diseases [101–104]. With the rise of RNA sequencing (RNA-Seq) technology, the number of lncRNAs identified has increased rapidly [105]. However, most lncRNAs have not been well annotated, and their regulatory mechanisms remain elusive. Furthermore, many lncRNAs are not evolutionarily conserved [106]. Therefore, it is vital to study the key function of the conserved lncRNAs.

## 4. circRNA and HCC

In addition to miRNA and lncRNA, other ncRNAs also influence the development of HCC [67, 107]. Circular RNAs (circRNAs) were discovered in mouse testes in 1993 [108] and represent another class of endogenous, noncoding RNA. In addition to being a biomarker for HCC, circRNA has recently been found to be an important gene expression and pathological network regulatory factor [109–111].

**Figure 1:**
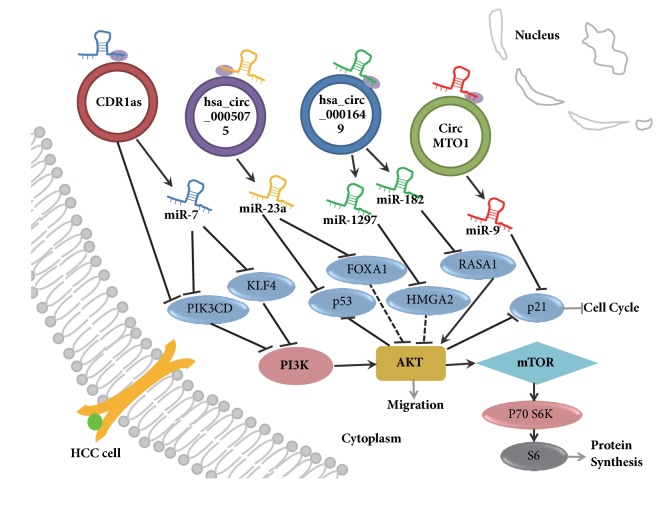
circRNAs function as microRNA sponges by targeting related genes.

The interactions of hsa_circ_0005075-targeted miRNA genes, including miR-23b-5p, miR-93-3p, miR-581, miR-23a-5p, and their corresponding mRNA, have been studied [70]. One circRNA, Cdr1as, inhibits and absorbs microRNA-7 (miR-7), a suppressor of HCC [69]. CircMTO1 (hsa_circRNA_0007874/ hsa_circRNA_ 104135) suppresses HCC progression by absorbing oncogenic miR-9, thus promoting p21 expression [68]. Recently, circrna_100338 was identified as a biomarker for HCC diagnosis and target for HCC therapeutics [67]. circRNAs function as microRNA sponges by targeting related genes shown in Figure 1 (modified from [108]) and Table 3. In addition, circ_0067934 directly inhibits miR-1324 that targets the 3′-UTR of FZD5 mRNA. Subsequently, the Wnt/*β*-catenin signaling pathway becomes downregulated in HCC, which enhances the proliferation, migration, and invasion of HCC [112].

## 5. snoRNA and Their Host Genes in HCC

Small nucleolar RNAs (snoRNAs) are a novel molecular species that may have significant influence on the development and progression of HCC [78]. The expression of snord113-1 in HCC is significantly downregulated [72]. The reduction of snord113-1 in HCC was clearly associated with decreasing patient. In essence, snord113-1 functionally inhibits the growth of HCC cells [72]. Small nucleolar RNA host gene 20 (SNHG20) expression in sk-hep-1 cells significantly inhibited cell proliferation, migration, and invasion. This suggests that SNHG20 could be used as an independent prognostic predictor for HCC patients [74]. The potential roles of snoRNA or their host genes in liver cancer are listed in Table 4.

## 6. Conclusion

This review provides an overall view of ncRNA in hepatocellular carcinoma (HCC). In addition to the recent focus of research towards lncRNA and miRNA, other ncRNAs, including circRNA and snoRNA also influence liver cancer. Although the first miRNA was identified 20 years ago, other ncRNAs including lncRNAs, snoRNA, siRNA, and piRNAs were gradually discovered and proved to be important in the pathogenesis of cancer [113]. To fully grasp the complete picture of the role of ncRNAs in the pathogenesis of HCC, it is paramount to explore the regulatory networks including circRNA-mRNA, miRNA-lncRNA, lncRNA/snoRNA-piRNA, and other networks that have not been found. To fully understand the biological functions of ncRNAs, we must ascertain the functions of all the proteins and ncRNAs of each cell type and the interactions among them. This full understanding is still a long way off, far more difficult than the genome project.

## Figures and Tables

**Table 1:** The function of abnormal expression miRNAs in HCC and in the serum of HCC patients.

miRNAs	Target gene	Function	References
miR-224	HOXD10	Promoting the invasion and metastasis of cancer cells	[43]
miR-224	SMAD4	Promoting cell proliferation	[44]
miR-224	ppp2r1b	Increasing the risk of liver cancer	[45]
miR-221	p27 and p57	Promoting the progression of hepatocellular carcinoma cell cycle	[46, 47]
miR-221	DDIT4	Promoting tumor development	[48]
miR-221	SOCS1 and SOCS3	Enhancing the effectiveness of interferon against HCV	[50]
miR-21	map2k3	Promoting the proliferation of cancer cells	[51]
miR-21	PDCD4	Increased the risk of liver cancer invasion and metastasis	[52]

**Table 2:** Signaling pathways in HCC regulated by lncRNAs.

lncRNAs	Pathways	Functions	References
URHC	ERK/MAPK pathway	Regulates cell proliferation and apoptosis	[53]
HULC	RXRA pathway	Modulates abnormal lipid metabolism	[54]
LINC00152	mTOR pathway	Promotes proliferation in HCC	[55]
LINC01225	EGFR-dependent pathway	Promotes occurrence and metastasis of HCC	[56]
CCHE1	ERK/MAPK pathway	Promotes carcinogenesis of HCC	[57]
linc-cdh4-2	R-cadherin pathway	Inhibits the migration and invasion of HCC cells	[58]
lncARSR	PTEN-PI3K/Akt Pathway.	Promotes doxorubicin resistance in HCC	[59]
TSLNC8	Interleukin-6/STAT3 pathway	A tumor suppressor	[60]
PDIA3P1	p53 pathway	Promotes cell proliferation, migration and invasion, and suppresses apoptosis	[61]
lncRNA00673	Notch pathway	Promotes of proliferation and metastasis of HCC	[62]
Igf2as	ERK/MAPK pathway	Controls hepatocellular carcinoma progression	[63]
CCAL	Wnt/*β*-catenin pathway	Promotes HCC progression	[64]
LncDQ	EMT Pathway	Promotes HCC progression	[65]
lnc-Myd88	NF-*κ*B and PI3K/AKT pathways	Promotes HCC progression	[66]

**Table 3:** circRNAs function as microRNA sponges in HCC.

circRNA	microRNA sponges	Cells	References
circRNA_10033	miR-141-3p	HCC and MHCC97H	[67]
circMTO1	miR-9	HCC	[68]
CDR1as	miR-7	HCC cells, SMMC-7221, Hep3B, QGY-7703, and HepG2	[69]
hsa_circ_0005075	miR-23b-5p, miR-93-3p, miR-581, miR-23a-5p	HCC cells, HepG2, and SNU449	[70]
hsa_circ_0001649	miR-1297	HCC cells, HepG2, and SMMC7721	[71]

**Table 4:** Potential role of snoRNA or their host genes in liver cancer.

SnoRNA/ their host genes	Potential role in liver cancer	Cells	References
SNORD113-1	A tumor suppressor role in HCC and a potential diagnostic and therapeutic target for HCC.	HepG2 and Huh7	[72]
SNHG3	Associated with malignant status and poor prognosis in HCC patients.	HCC	[73]
SNHG20	Up-regulated in patients with HCC, served as an independent prognostic predictor for HCC patients.	HCC and SK-Hep-1	[74, 75]
SNHG1	A prognostic biomarker and therapeutic target for HCC.	HCC and HepG2	[76, 77]
SNORD78	Associated with aggressive phenotype and poor prognosis of HCC	SK-Hep-1	[78]
SNHG6	Impacts HCC tumorigenesis by binding to up-frameshift protein 1 and regulating Smad7 expression.	HCC and L02	[79]
SNORD126	A therapeutic target	HepG2, LS174T and Huh7	[80]
SNHG12	A biomarker and a potential therapeutic target for HCC.	HCC	[81]
ACA11	A promising prognostic biomarker and therapeutic target for patients with HCC.	HCCLM9 and SK-Hep1	[82]
snoRA47	A valuable biomarker and a potential therapeutic target for HCC.	HCC	[83]
SNORD76	A promising prognostic biomarker in patients with HCC.	SK-Hep1 and Huh7	[84]
